# Investigating the Synergistic Potential of Low-Dose HDAC3 Inhibition and Radiotherapy in Alzheimer’s Disease Models

**DOI:** 10.1007/s12035-023-03373-0

**Published:** 2023-05-12

**Authors:** Natalie R. Ricciardi, Farzaneh Modarresi, Ines Lohse, Nadja S. Andrade, Ian R. Newman, Jonathan M. Brown, Caroline Borja, Brian Marples, Claes R. Wahlestedt, Claude-Henry Volmar

**Affiliations:** 1 Department of Biochemistry and Molecular Biology, University of Miami, Miami, FL 33136 USA; 2 Department of Psychiatry and Behavioral Sciences, University of Miami, Miami, FL 33136 USA; 3 Center for Therapeutic Innovation, University of Miami, Miami, FL 33136 USA; 4 Department of Radiation Oncology, University of Miami, Miami, FL 33136 USA

**Keywords:** HDAC inhibitor, Radiation, Low-dose, Alzheimer’s disease, Cognition

## Abstract

We have previously shown that histone deacetylase (HDAC) inhibition and cranial radiotherapy (RT) independently improve molecular and behavioral Alzheimer’s disease (AD)-like phenotypes. In the present study, we investigate the synergistic potential of using both RT and HDACi as a low-dose combination therapy (LDCT) to maximize disease modification (reduce neuroinflammation and amyloidogenic APP processing, increase neurotrophic gene expression) while minimizing the potential for treatment-associated side effects.

LDCT consisted of daily administration of the HDAC3 inhibitor RGFP966 and/or bi-weekly cranial x-irradiation. Amyloid-beta precursor protein (APP) processing and innate immune response to LDCT were assessed *in vitro* and *in vivo* using human and murine cell models and 3xTg-AD mice. After 2 months of LDCT in mice, behavioral analyses as well as expression and modification of key AD-related targets (Aβ, tau, Csf1r, Bdnf, etc.) were assessed in the hippocampus (HIP) and prefrontal cortex (PFC).

LDCT induced a tolerant, anti-inflammatory innate immune response in microglia and increased non-amyloidogenic APP processing in vitro. Both RT and LDCT improved the rate of learning and spatial memory in the Barnes maze test. LDCT induced a unique anti-AD HIP gene expression profile that included upregulation of neurotrophic genes and downregulation of inflammation-related genes. RT lowered HIP Aβ_42/40_ ratio and Bace1 protein, while LDCT lowered PFC p-tau181 and HIP Bace1 levels.

Our study supports the rationale for combining complementary therapeutic approaches at low doses to target multifactorial AD pathology synergistically. Namely, LDCT with RGFP966 and cranial RT shows disease-modifying potential against a wide range of AD-related hallmarks.

**Supplementary Information:**

The online version contains supplementary material available at 10.1007/s12035-023-03373-0.

## Background

Alzheimer’s disease (AD) is a multifactorial disease characterized by numerous pathologies including increased neuroinflammation, decreased synaptic plasticity, dysregulated amyloid precursor protein (APP) processing, and aberrant tau protein post-translational modifications [1]. Single-target therapeutic approaches have yielded suboptimal results over the last 2 decades in efforts to slow or reverse cognitive decline in AD. Currently approved small molecule compounds are not disease-modifying, and the effects of more recent anti-amyloid immunotherapies on cognitive function are controversial [2–4]. Here, we pursue two nontraditional therapeutic approaches individually and in combination: (1) inhibition of the epigenetic enzyme histone deacetylase 3 (HDAC3) via low-dose RGFP966 and (2) low-dose cranial radiotherapy (RT).

Epigenetic drugs are attractive therapeutic agents in complex diseases due to pleiotropic downstream effects through selective targeting of a single enzyme or class of enzymes [5]. Specifically, histone acetylation is essential to long-term memory formation and is found to be dysregulated in the prefrontal cortex (PFC) of AD post-mortem brains [6, 7]. We and others have demonstrated that class I HDAC inhibitors (HDACi), including the HDAC3-selective small molecule RGFP966, have disease-modifying potential when administered systemically in AD mouse models [8–10]. Additionally, genetic silencing and overexpression of HDAC3 in the hippocampus of transgenic AD mice supports the role of HDAC3 as a negative regulator of memory [11–13]. While traditional investigation of the mechanism of action of HDAC3 suppression is centered around histone acetylation, recent research has revealed that HDAC inhibition dampens neuroimmune signaling, but this mechanism is relatively underexplored in AD [14–17]. Furthermore, advancement of HDACi into AD clinical trials has been hindered by known hematological side effects observed in clinical cancer studies which employ relatively high doses. Subthreshold (non-toxic) doses, isoform-specific HDACi, and the anti-neuroinflammatory potential of HDACi have not been clinically pursued in AD [18].

Low-dose radiation has been explored as a noninvasive therapeutic option for a wide range of diseases with amyloidosis as a featured pathology [19, 20]. While demonstrated to be effective in many of these contexts, recent applications in AD animal models using repeated fractions of 0.5–2 Gy of x-irradiation have revealed benefits including reduced amyloid plaque load, reduced phosphorylated tau (p-tau), resistance to neuroinflammatory conditions, and increased neuron viability in concert with improved cognitive performance [21–26]. In addition, these low doses benefit brain physiology with none of the expected detriments associated with high doses of radiation. Many critical aspects of cranial radiotherapy for AD, including mechanism, dose, and dosage regimen, remain elusive.

Recent attention has been shifted to anti-neuroinflammatory approaches in the development of drugs against AD, partly due to the fact that a majority of all AD genetic risk loci are enriched in microglia [27]. Despite this, the immunomodulatory effects of radiation and HDACi have not been explored in the context of AD [21, 23, 28]. How these treatments affect cytokine production (e.g., *Il1b*, *Il6*, *Il10*, and *Tnfα)* or master regulators of glial function (e.g., *Spi1*, *Iba1*, *Csf1r*, and *Trem2)* is not well characterized. Microglia, the innate immune cells of the brain, exist in a hyperinflammatory state in AD animal models and human post-mortem tissue and have been shown to exacerbate the diseased state in response to tau and amyloid pathology [29]. This is evidenced by changes in the cytokine profiles of microglia along with modified interaction between microglia and amyloid/tau and other CNS cell types. Microglial states can become polarized in response to single or repeated exposures to a stimulus, exhibiting either a trained (hyperinflammatory) or tolerant (anti-inflammatory) phenotype, respectively [30]. Mounting evidence suggests that both low-dose radiation and HDAC inhibition have the potential to promote the tolerant microglial phenotype which is resistant to inflammatory stimuli, including oligomeric amyloid-beta (Aβ) which is hypothesized to be the most neurotoxic form of Aβ [23, 31, 32].

Here, we examined the synergistic potential of low-dose combination therapy (LDCT) which consists of cranial radiotherapy (RT) and systemic administration of RGFP966 in aged 3xTg-AD mice. We found that the combination of the two treatments was synergistic and created a unique gene expression profile that includes improved neurotrophic signaling and downregulation of genes associated with activated microglia. In addition, modification of amyloid pathology in the 3xTg-AD model and improved spatial memory in the Barnes maze were observed.

## Methods

### Cell Culture

SIM-A9 (CRL-3265) spontaneously immortalized murine microglial cells and Neuro-2a (N2A, CCL-131) murine neuroblast cells were acquired from American Type Culture Collection (ATCC). HEKAPP_Swe_ cells were kindly provided by the Selkoe laboratory (Brigham and Women’s Hospital, Harvard Medical School). HEKAPP_Swe_ cells contain a stably incorporated overexpression vector for APP with the Swedish double mutation (K670N/M671L). This mutation shifts APP processing toward a more amyloidogenic state, increasing total levels of Aβ_40_ and Aβ_42_ [33]. SIM-A9 culture media: DMEM/F12 (Gibco), 10% heat-inactivated (HI) FBS (Gibco), 5% horse serum (Gibco), and 1X Pen/Strep (Gibco). HEKAPP_Swe_ culture media: DMEM/F12, 10% HI FBS, 100 μg/mL Primocin (InvivoGen), and 250 μg/mL G-418 selection reagent (Gibco). N2A culture media: mix 50% DMEM and Opti-MEM (Gibco), 5% HI FBS, and 1% Pen/Strep. Cells were cultured in a sterile environment under 5% CO_2_ at 37 °C and tested for mycoplasma monthly.

### Cytotoxicity Assay

Cytotoxicity of different HDAC inhibitors was evaluated using the CellTiter-Glo luminescent cell viability assay from Promega, as per the manufacturer’s instructions. Briefly, SIM-A9 cells were seeded overnight in a 384-well plate, treated for 48 h with each compound or DMSO control, and ATP was measured after treatment using the CellTiter-Glo reagent. Since only live metabolically active cells produce ATP, this assay allows the measurement of viable cells in each well. Data are presented as percent of Velcade response (positive control).

### In Vitro Combination Therapy

On day 1, cells were plated at a density of 2 × 10^5^ cells/well (SIM-A9) or 5 × 10^5^ cells/well (HEKAPP_Swe_) in a 12-well plate with 3 biological replicates per treatment group. On day 2, cells were treated via media exchange with either 3 μM RGFP966 or DMSO followed by x-irradiation in the RS225 Xstrahl Cabinet Irradiator with the following parameters: 195 kV and 0 mA with a 0.5 mm Cu filter at 500 mm FSD over 100s. The experiment was ended at the specified time point by adding either mammalian protein extraction reagent (M-PER, ThermoFisher) supplemented with 2X protease and phosphatase inhibitors, TRIzol (Invitrogen), or RLT buffer (Qiagen) supplemented with β-mercaptoethanol (Sigma).

#### Cellular Protein Extraction

Cells were washed with PBS, aspirated, and lysed with protease and phosphatase inhibitor supplemented M-PER. Plates were then frozen at −80 °C for at least 15 min. Plates were then allowed to thaw on ice or rocking at 4 °C. Cell scrapers (Falcon) were used to lift the cells, and the cell suspension was transferred to microfuge tubes where they sat on ice for 30 min. Samples were centrifuged at 10,000 × g for 10 min at 4 °C, and the supernatant was transferred off the cell pellet for further protein analysis. Concentrations of protein samples were determined with the Pierce BCA Protein Assay Kit (Thermo Fisher Scientific) following the manufacturer’s protocol. Spectroscopy measurements were made at 560 nm (EnVision Multilabel Plate Reader, Perkin Elmer Inc.). Sample protein concentrations were interpolated off a second-order polynomial generated from the protein standards.

#### Cellular RNA Extraction

Cells were lysed with RLT buffer and subsequently processed on a column following the protocol of the RNeasy kit (Qiagen) with the optional DNase digestion step included. Alternatively, TRIzol (Thermo Fisher Scientific) was used for RNA extraction according to the manufacturer’s protocol but with the addition of GlycoBlue (Invitrogen) co-precipitant and DNase digestion (TURBO DNA-free, Invitrogen). RNA concentration and purity was determined via spectroscopy on the NanoDrop (Thermo Fisher Scientific).

### Training vs. Tolerance Assay

To assess how LDCT impacts innate immune response to Aβ, 48-h treated SIM-A9 cells were stimulated with conditioned media derived from N2A cells overexpressing APP_Swe/Ind_. pCAX APP Swe/Ind was a gift from Dennis Selkoe & Tracy Young-Pearse (Addgene plasmid # 30145; http://n2t.net/addgene:30145; RRID:Addgene_30145). The Swedish and Indiana familial AD mutations combined elevate the Aβ_42/40_ ratio by increasing amyloidogenic processing of APP at the β-secretase and γ-secretase cleavage sites, respectively [34]. On day 1, SIM-A9 cells were plated 5 × 10^4^ in a 12-well plate with 3 biological replicates per treatment group. On day 2, 5 × 10^6^ N2A cells were reverse transfected in a T75 flask with 30 μg of the APP_Swe/Ind_ plasmid in 15 mL of media using Lipofectamine 3000 (Invitrogen). Additionally, the SIM-A9 cells received LDCT. An empty pCAX vector identical to the APP_Swe/Ind_ backbone was used as the control. On day 4 (48 h after SIM-A9 treatment and N2A transfection), N2A media from transfected and control flasks was collected and centrifuged to remove any dead cells (300 RPM, 3 min at RT) before stimulating the SIM-A9 cells with the conditioned media for 4 h. Media was collected, centrifuged, and the supernatant was isolated to quantify Aβ levels (Fig. S1I). Cells were washed with PBS before adding TRIzol and frozen at −80 °C for RNA extraction.

### Aβ Uptake Assay

#### Primary Microglia Isolation

We utilized a lightly modified version of the protocol for the Miltenyi Biotec MACS cell separation system to isolate adult primary microglia. Following a lethal dose of isoflurane and cervical dislocation, brains from two 14-month-old male C57Bl/6J mice were isolated under a biosafety cabinet to maintain a sterile environment. The brainstem, olfactory bulbs, and cerebellum were removed before manual dissociation of the brain in PBS and transferring the brain to a 15 mL tube for enzymatic digestion with papain according to manufacturer protocol. After straining the cell suspension through a 70 μm strainer, myelin removal was performed by resuspending the pelleted cells in 8 mL of 30% Lymphoprep (Stem Cell Technologies) in DMEM. A total of 2 mL of 70% Lymphoprep in PBS was then underlayed in the bottom of the 15 mL tube before centrifuging at 300 g for 5 min. at 4°C. The supernatant was removed, and the middle layer was collected for labeling with CD11b beads and magnetic separation on MACs LS columns according to the manufacturer’s protocol (Miltenyi Biotec).

#### Aβ Uptake Assay

On day 1, 2 × 10^5^ primary microglia were plated onto PDL-coated 2-chamber glass slides in DMEM/F12 containing 10% FBS, 1% GlutaMAX (Gibco), 1% sodium pyruvate, 1% NEAA (Gibco), and 1% Pen/Strep. On day 2, 1/2 of the media was changed. On day 3, cell media was completely replaced, and cells were treated with RGFP966 and/or RT. On day 4, oligomeric Aβ_42_-555 (Anaspec) was prepared by dissolving 0.0225 mg of Aβ_42_-555 in 21 uL of DMSO and sonicating for 2 min. This solution was then diluted into culture media at 300 nM and left to oligomerize overnight in a 37 °C incubator. On day 5 (48 h after LDCT treatment), culture media was replaced with media containing Aβ_42_-555 and incubated for 2 h before washing 3 X PBS and fixing cells with 4% paraformaldehyde in PBS in preparation for immunocytochemistry (ICC).

### Animals and Treatment

Fourty 3xTg-AD mice (MMRRC Strain #034830-JAX) were obtained from The Jackson Laboratory (1:1 M:F ratio). These mice contain both the Psen1^tm1Mpm^ and Tg (APPSwe, tauP301L)1Lfa alleles under control of a CNS-enriched Thy1 promoter on a congenic C57BL/6J background. All experiments were approved by the University of Miami Miller School of Medicine Institutional Animal Care and Use Committee and conducted according to the specifications of the NIH as outlined in the Guide for the Care and Use of Laboratory Animals. Mice were group-housed (3–5 per cage) in standard shoebox cages with bedding and nesting materials, located in ventilated racks in the rodent vivarium. Throughout the study, mice had *ad libitum* access to food and water and were maintained on a standard 12 h light-dark cycle (lights on 600–1800 h).

Beginning at 9 months of age, 3xTg-AD mice were treated for 2 months with intraperitoneal injections of either 3 mg/kg RGFP966 or saline 5 days/week and either 1 Gy of cranial x-irradiation or sham irradiation 2 days/week. Mice were randomly assigned to treatment groups to avoid cage effects. Radiotherapy sessions were administered 3–4 days apart for a total dose of 16 Gy. RGFP966 stock solution was made in 100% DMSO at 50 mg/mL. The drug solution was prepared fresh each day consisting of 5% tween-80 in 0.9% sterile saline. RGFP966 or DMSO was added to create a 1mg/mL RGFP966 or 2% DMSO (vehicle) solution for administration via intraperitoneal injection. On radiation days, the drug was administered immediately before being placed in the radiation cabinet.

Cranial radiotherapy was administered under 1–3% isoflurane anesthesia (balance 100% O_2_) for immobilization and held on 0.4% isoflurane with a multi-animal breather (World Precision Instruments) during irradiation to maintain treatment precision. Lead shielding was placed over the immobilized bodies starting immediately behind the skull. After a short warm-up, a dose of 1 Gy x-irradiation was administered in the RS225 Xstrahl Cabinet Irradiator with the following parameters: 195 kV and 10 mA with a 0.5 mm Cu filter at 500 mm focal surface distance (FSD) with a dose rate of 0.52 Gy/min, 1st and 2nd half-value layer = 1.01 and 1.76 mm Cu, respectively. Sham-irradiated animals were placed in the closed cabinet for the same amount of time under isoflurane anesthesia. After completion of treatment, mice aged for an additional month without treatment before behavioral assessment. Spatial memory was assessed via Barnes maze (BM) and Y-maze (YM). Working memory was tested using novel object recognition (NOR).

### Behavioral Testing and Data Analysis

#### Open Field (OF)

To examine the effects of treatment on locomotor activity, mice were individually placed in the center of an open field arena (27 × 27 × 23 cm) in a quiet, well-lit room for 10 min. Horizontal activity was detected using a ceiling-mounted camera and Ethovision (Noldus) automated tracking software, and the total distance as well as the distance that each mouse traveled over that time period were recorded. All arenas were cleaned with 70% ethanol between mice.

#### NOR

Two identical grey cubes (4 x 4 x 4 cm) were placed in the OF arena, and mice were allowed to explore the objects for 5 min (training period). After a 24-h intertrial interval, one of the objects was replaced with a white sphere, and the mouse was allowed to explore for 3 min (testing period). This test is based on the spontaneous tendency of rodents to spend more time exploring a novel object than a familiar one. Exploration of the novel object reflects the use of learning and recognition memory. Ethovision was used to quantify the duration of mouse nose spent in object zones 1.5 cm around the objects, and a discrimination index (DI) was calculated: (duration in novel zone – duration in familiar zone) / (total time in either zone).

#### YM

The Y-maze spontaneous alternation test measures exploratory behavior based on the willingness of the mice to visit a new arm of the maze rather than a familiar arm. It is a test of hippocampal function but also includes use of other parts of the brain such as the septum, basal forebrain, and prefrontal cortex. The apparatus we used consisted of three, equally well-lit enclosed arms (30-cm length, 5-cm width, and 10-cm height) in the shape of a Y. Mice were randomly placed in a start arm of the Y maze. Upon leaving the start arm, the mouse chooses between entering either the left or the right goal arm. With repeated trials, a mouse with no cognitive impairment typically shows less of a tendency to enter a previously visited arm. The spontaneous alternation percent (SAP) as per Arendash et al., with the formula: SAP = 100 × number of alternation/(total arm entries −2) [35].

#### BM

The Barnes maze protocol was adapted from Attar et al. and used to assess differences in long-term spatial and working memory [36]. Five training trials of 3 min/trial were performed over 3 days. In addition to the naturally existing spatial cues of the room, 4 large shapes of variable color were printed on white paper and placed in the 4 directions of the room. Mice were placed under an opaque box for 10 s in the center of the field. The box was lifted, and the 3-min trial began. The mouse searched for the target hole where a dark cubby with bedding material was placed in the same location in the room relative to the spatial cues. After each trial, the table was rotated to eliminate the possibility of any scent cues or table markings as spatial cues. After a 48-h break from training, mice were again placed in the center of the field and allowed to explore for 3 min, but the cubby was removed. Four parameters were assessed: primary latency to target hole, errors to target hole, time spent in the target quadrant, and % of total holes searched in the target quadrant.

### Tissue Collection and Processing

Mice were subjected to a lethal dose of isoflurane (> 5%), and blood was collected via cardiac puncture with a 25G 5/8” needle attached to a 1 mL syringe in 1.5 mL tubes with 0.5M EDTA on ice. After all animals were euthanized, whole blood was centrifuged at 1500 g for 5 min at 4 °C, and plasma was collected and frozen at −80 °C. Tissue was snap-frozen on dry ice and stored in −80 °C for further processing.

#### Tissue Processing

The mirVana PARIS kit (Ambion) was used for protein and RNA extraction of the hippocampus according to the manufacturer’s protocol. Briefly, 1/2 of the hippocampus (~20 mg) was homogenized in 250 μL of cell disruption buffer supplemented with 1X protease inhibitor (HALT^TM^ protease inhibitor cocktail 100X, ThermoFisher), 1X phosphatase inhibitor (Halt™ Phosphatase Inhibitor Cocktail, ThermoFisher), and RNase inhibitor (SUPERase●In, Invitrogen). Homogenization was performed in the MM 400 (Retsch) with 1 5 mm stainless steel bead (Qiagen) per 250 μL sample at a frequency of 30 Hz for 30 s. The sample was then split in half for final protein and RNA isolation.

#### Tissue Protein Extraction

A total of 125 μL lysate was further sonicated in the supplemented cell disruption buffer for 30 s intervals on medium frequency for 2 min (BioRupter UCD-200). After a 10-min incubation on ice, the lysate was centrifuged at max speed for 1.5 min at 4 °C, and the soluble supernatant was collected and used for ELISA and western blotting. PFC protein was extracted using M-PER (ThermoFisher) supplemented and homogenized identical to lysate in the mirVana PARIS protocol.

#### Tissue RNA Extraction

Hippocampal RNA was extracted from the remaining 125 μL lysate according to the mirVana PARIS protocol using a column-based approach. The protocol was modified to include DNase digestion on the final column eluent (TURBO DNA-free, Invitrogen). RNA was quantified using nanodrop and Qubit RNA HS Assay Kit (Thermo Scientific) and used for RT-qPCR and NanoString gene expression assays.

#### Acid Extraction of Histones

A total of 20–40 mg of cortex tissue was homogenized in 300 uL of tissue extraction buffer (0.5% Triton X-100, 1 mM PMSF, 0.02% NaN_3_, 100 nM SAHA, 1X PBS) using BioMasher II Tissue Homogenizers. Samples were then frozen and thawed before pelleting (10,000 g, 7 min, 4 C) and removing the supernatant. The pellet was resuspended in hypotonic lysis buffer (10 mM Trix-HCl pH 8, 1 mM KCl, 1.5 mM MgCl_2_, 1 mM DTT, 1X protease inhibitor cocktail, 1X phosphatase inhibitor cocktail, and 100 nM SAHA) and rotated at 4 C for 30 min. The nuclei were pelleted (10,000 g for 10 min, 4 C), and the supernatant was discarded. Histones were extracted by resuspending the pellet in 0.4 M H_2_SO_4_ and rotating at 4 C 30 min-12 h. Insoluble non-histone proteins were removed by pelleting (16,000 g for 10 min, 4 C). The supernatant was transferred to a new tube, and histones were precipitated out with the addition of 264 uL 50% trichloroacetic acid (TCA) and rotating at 4 C for at least 1 h. Crude histones were pelleted (16,000 g for 10 min, 4 C), and the pellet was washed 2X with ice-cold 100% acetone before air drying and resuspending in Milli-Q water. The pellet was sonicated in water to help dissolve histones, re-pelleted, and the final supernatant was collected and quantified using a Bradford assay (Bio-Rad).

### Western Blotting

A total of 30 μg of soluble protein lysate with XT Sample Buffer (BioRad) containing β-mercaptoethanol was boiled at 100 °C for 10 min. Precision Plus Protein Dual-Color Standards (BioRad) and boiled samples were added to wells of a 26-well 4-12% Criterion^TM^ XT Bis-Tris Protein Gel (BioRad) and run at 150 V for 45 min in XT MES buffer. Protein was transferred onto 0.2 μm PVDF membranes using the Trans-Blot Turbo Transfer Pack (BioRad). Blots were incubated, rocking for 1 h at room temperature with 5% Blotting-Grade Blocker (BioRad) in 1X TBS (BioRad) with 0.1% TWEEN20 (TBST). Following three 5-min TBST washes, primary antibody was added. After overnight incubation at 4 °C, blots were washed 3 × 5 min in TBST before secondary antibody in 5% blotting grade blocker for 1 h at RT. Clarity Western ECL Substrate (BioRad) or SuperSignal West Femto Maximum Sensitivity Substrate (Thermo Scientific) were used to expose blot signal (LI-COR Odyssey DLx).

### Gene Expression

Relative abundance of RNA species was determined using RT-qPCR. A total of 2 μg of RNA from samples were reverse transcribed into cDNA using the qScript cDNA Synthesis Kit (QuantaBio). A total of 100 ng of cDNA was then added to wells in a 384-well format along with TaqMan Fast Advanced Master Mix (Thermo Scientific) and the desired TaqMan probe/primer, with each reaction in technical duplicate. Plates were then loaded into a QuantStudio Real-Time PCR machine, and the TaqMan Fast Advanced program was initiated (Applied Biosystems). The ddCt method was used to analyze the output [37]. Briefly, we calculated the differences between the Ct values for target and reference genes (Rpl37a) as ΔCt and the difference between the resulting ΔCt and that of the vehicle control (calibrator sample) to obtain the ΔΔCt. Results are presented as fold change (RQ = 2^−ΔΔCt^) for mRNA expression relative to the vehicle. Any target with Ct values above 32 were not analyzed or repeated with a higher concentration of cDNA. All genes tested by qPCR in these studies were amplified with Taqman primers from Life Technologies/Thermo Fisher Scientific.

We also performed a medium-throughput transcriptomic analysis using the NanoString Technologies nCounter® Mouse Neuropathology Panel which includes 770 genes specific for neurodegeneration and 18 additional custom targets. Quality control of input HIP RNA was performed on the Agilent Bioanalzyer prior to expression profiling on the nCounter SPRINT according to the manufacturer’s instructions. Advanced analysis was performed within the nSolver software that included QC of raw counts, count normalization to 10 housekeeping genes, and Gene Set Analysis (GSA). DEGs were identified by those with a *p*-value < 0.05. GSA results, similar to Gene Ontology, are explained by directed global significance scores which measure the tendency to have over- or under-expressed genes within a given Gene Set and are derived from the DEGs in that set.

### ELISAs

ELISAs were performed according to the manufacturer’s instructions to obtain a relative quantity of Aβ_40_, Aβ_42,_ p-tau181, p-tau396, and total tau (ThermoFisher). Data presented is % control after calculating either Aβ_42/40_ ratio or normalizing p-tau data to total tau. Briefly, soluble protein or centrifuged culture media was diluted with kit diluent according to the table below:

### Statistical Analyses

All data are represented as mean ± SEM, and sample size is reported in figure legends. Group means ± SEM and sample sizes (*n*) are reported in each figure legend. Data were statistically significant if *p* < 0.05. For all figures, all statistically significant group differences are labeled. For any given group comparison, the lack of any indication of significant difference implies a lack of significance by the applied statistical test. GraphPad Prism software was used for all statistical analyses except those associated with the NanoString assay. Outlier evaluation for all statistical tests was performed using the ROUT method (*Q* = 1%) in GraphPad Prism

## Results

### LDCT Elicits a Tolerant Innate Immune Response in Microglia

**Fig. 1:**
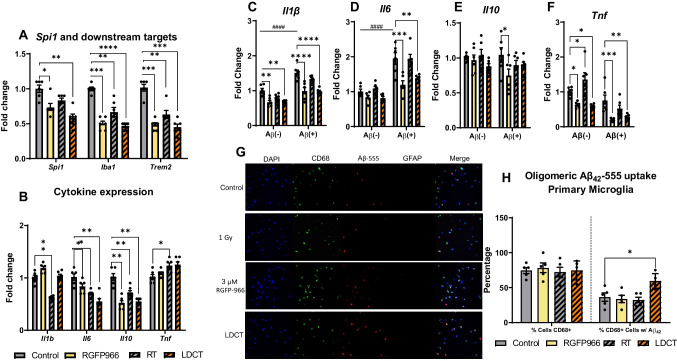
48 h LDCT downregulates cytokine expression and induces a tolerant microglial phenotype. **A** RT-qPCR reveals RGFP966 and RT independently and combined downregulate expression of microglial regulator genes *Spi1*, *Iba1*, *and Trem2* in SIM-A9 cells. **B** RGFP966, RT, and LDCT downregulate *Il6* and *Il10* gene expression while only RT downregulates *Il1b* gene expression and modestly upregulates *Tnfa* gene expression in SIM-A9 cells. **C–F** After 48 h treatment, stimulation with conditioned media containing Aβ_42_ induced an increase in *Il1b* and *Il6* gene expression that was blocked in RGFP966 and LDCT pre-treated cells. Conditioned media did not elicit an increase in *Il10* or *Tnfa* gene expression in control-treated cells; however, stimulation with conditioned media decreased *Il10* gene expression in RGFP966 pre-treated cells and *Tnfa* in RGFP966 and LDCT pre-treated SIM-A9 cells. **G** Representative image and **H** quantification of primary microglial uptake of oligomeric fluorescent Aβ_42_-555. 48 h LDCT pre-treated CD68+ primary microglia culture had greater Aβ_42_-555 % positivity than control or individually treated cells. Data are represented as mean ± SEM (RT-qPCR: *n* = 6 biological replicates, microscopy: *n* = 5 frames containing 100+ cells). **A**, **B**, **H** One-way ANOVA. **C**–**F** Two-way ANOVA. Dunnett’s multiple comparison test *P<0.05, **P<0.01, ***P<0.001, ****P<0.0001. # denotes statistical significance when comparing between Aβ(-) and Aβ(+) groups

We first investigated the immunomodulatory potential of low-dose RGFP966 (3 μM), low-dose RT (1 Gy), and their combination (LDCT) in immortalized mouse microglia (SIM-A9) and primary murine microglia after 48 h of treatment. LDCT in SIM-A9 microglia induced a 1.72-fold decrease (*P* < 0.0001) for the microglia-enriched master transcription factor *Spi1* (PU.1 in humans) and two downstream targets *Iba1* (2.13-fold, *P* < 0.0001) and *Trem2* (2.22-fold, *P* < 0.0001) as determined by RT-qPCR (Fig. 1A) [38]. We then examined gene expression of commonly indicated cytokines in AD and found treatment-dependent depression of *Il6* and *Il10* expression but no significant effects on *Tnfα* expression (Fig. 1B). Specifically, RT alone significantly decreased *Il1β* expression (1.61-fold, *P*<0.0001) but not LDCT. LDCT treatment induced a 1.82-fold decrease (*P*<0.0001) in both *Il6* and *Il10* gene expression. Temporal analysis of these genes at 12-, 24-, 48-, and 96-h timepoints revealed a dynamic response to LDCT whereby inflammatory gene expressions of targets *Spi1* (*P*<0.0001), *Iba1* (*P*<0.05), *Trem2* (*P*<0.0001), *Il10* (*P*<0.05), *and Il6* (*P*<0.05) were minimized at 48 h by LDCT treatment (Fig. S1A-G). However, LDCT had no significant effect on temporal expression of *Tnf* and *Apoe*, an innate immune regulator, throughout this time course [39]. We verified that changes in gene expression were not related to cell death as HDACi have been reported to be acutely myelosuppressive [40]. Compared to two other pan-HDACi, M344 and Quisinostat, RGFP966 did not affect microglial cell viability (Fig. S1H). Interestingly, innate immune gene expression followed a similar pattern in response to both RGFP966 and RT individual treatments during the time course, with additive effects often observed in response to LDCT.

This pattern of cytokine suppression at 48 h mimics the previously reported tolerant microglial phenotype which is resistant to inflammatory stimuli [30]. To investigate this tolerant phenotype, we stimulated 48-h treated SIM-A9 cells with conditioned media containing elevated levels of Aβ_42_ (Aβ+) or conditioned media from cells overexpressing an empty plasmid (Aβ-) (Fig. S1I). Gene expression analysis of the stimulated SIM-A9 cells revealed that (1) Aβ+ conditioned media significantly upregulated *Il1β* (1.51-fold, *P*<0.0001) and *Il6* (1.95-fold, *P*<0.0001) gene expression in control-treated cells compared to Aβ- media, and (2) this upregulation was blocked by pretreatment with LDCT. This supports the induction of a tolerant phenotype induced by LDCT (Fig. 1C, D). In contrast, neither LDCT pretreatment nor conditioned media had any effect on *Il10* expression (Fig. 1E). Additionally, Aβ+ conditioned media did not induce *Tnf* expression in control-treated cells, but pretreatment with RGFP966 or LDCT reduced *Tnf* expression under both stimulated conditions (Fig. 1F). To assess functional consequences of the tolerant microglial phenotype, we used confocal microscopy to measure uptake of fluorescently labeled, oligomeric Aβ_42_-555 in primary murine microglia. After microglia isolation, cells were stained with DAPI, CD68 (myeloid cell marker), and GFAP (astrocyte marker) to determine primary culture purity (Fig. 1G). Dual DAPI+CD68+ cells were equivalent across treatment groups (range: 72–78% pure microglia), and no contaminating astrocytes were observed in the primary microglia culture (Fig. 1H). After 48-h LDCT pretreatment followed by stimulation with oligomeric Aβ_42_-555, we observed a significant increase (1.64-fold, *P*<0.05) in triple positive DAPI+CD68+Aβ_42_-555+ cells only in the LDCT-treated cells compared to control, suggesting the LDCT increases microglial ability to uptake oligomeric Aβ_42_.

### LDCT Modifies APP Processing and Neurotrophic Gene Expression in Vitro

**Fig. 2:**
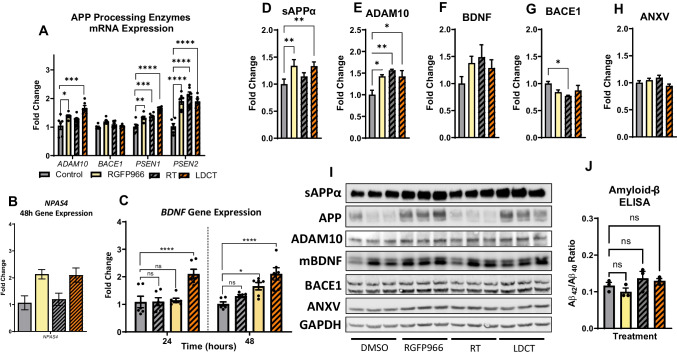
LDCT modulates APP processing and *BDNF* expression in HEKAPP_Swe_ cells. **A** RT-qPCR reveals RGFP966 and RT independently and combined upregulate expression of non-amyloidogenic APP processing enzymes *ADAM10*, *PSEN1*, and *PSEN2* at 48 h without affecting *BACE1* expression **B,C** RGFP966 and LDCT upregulate expression of neurotrophic genes *NPAS4* and *BDNF.* **D–I** Immunoblotting of sAPPα, ADAM10, mature BDNF (mBDNF), BACE1, and annexin V (ANXV) at 48 h. **J** ELISA reveals no change in Aβ_42/40_ ratio in HEKAPP_Swe_ supernatant after 48 h treatment. Data are represented as mean ± SEM (RT-qPCR: *n* = 6 biological replicates, immunoblotting: *n* = 3). One-way ANOVA using Dunnett’s multiple comparisons test *P<0.05, **P<0.01, ***P<0.001, ****P<0.0001

We then investigated the effects of LDCT on amyloid pathology *in vitro *by examining alterations in APP processing using the HEKAPP_Swe_ AD cell model [8, 41, 42]. The Swedish familial AD mutation causes an increase in both Aβ_40_ and Aβ_42_ production compared to wildtype APP [33]. APP is enzymatically processed by α-secretases (e.g., ADAM10, non-amyloidogenic), β-secretases (e.g., BACE1, amyloidogenic), and γ-secretases (e.g*.*, PSEN1/2) [43]. We observed a 1.67-fold increase (*P*<0.0001) in *ADAM10* gene expression in response to 48 h LDCT, while single treatments provided a modest upregulation of *ADAM10* (RGFP966: 1.38-fold, *P*<0.05 and RT: not significant) (Fig. 2A). Treatment with either RGFP966 or radiation alone increased the expression of γ-secretases *PSEN1* (1.32-fold, *P*<0.05 and 1.37-fold, *P*<0.01, respectively) and *PSEN2* (1.91-fold, *P*<0.0001 and 2.09-fold, *P*<0.0001, respectively). This upregulation was sustained in the LDCT treatment group with *PSEN1* showing a 1.63-fold increase (*P*<0.0001) and *PSEN2*, *a* 1.9-fold increase (*P*<0.0001). Expression of the main β-secretase for APP, *BACE1*, was unaffected by all treatments in these cells. We also observed an upregulation of gene expression for the transcription factor *NPAS4* (2.1-fold, *P*<0.01) an HDAC3-specific inducer of brain-derived neurotrophic factor (*BDNF*) (Fig. 2B). *BDNF* expression was elevated 1.93-fold only by LDCT at 24h (*P*<0.001) and stayed elevated at 48 h (1.92-fold, *P*<0.001) (Fig. 2C). This change was driven mostly by RGFP966 treatment alone (*NPAS4:* 2.13-fold*, P*<0.01 and *BDNF:* 1.57-fold, *P*<0.05) as RT alone had no significant effect on these gene targets. Immunoblotting revealed that LDCT induced a 1.33-fold increase (*P*<0.05) in production of soluble APPα (sAPPα, non-amyloidogenic processing fragment) and a 1.42-fold increase in ADAM10 expression (*P*<0.05) but no significant effect in BACE1 protein expression (Fig. 2C, D, F, H). All treatments induced slight upregulation of BDNF, but the data were not significant (Fig. 2E). We measured Annexin V (ANXV) to observe any potential apoptotic effects of the treatments with no significant differences detected (Fig. 2G). Lastly, we did not observe a change to the Aβ_42/40_ ratio *in vitro* after 48 h of treatment (Fig. 2I).

### LDCT Induces Modest Spatial Memory Improvements in 3xTg-AD Mice

**Fig. 3:**
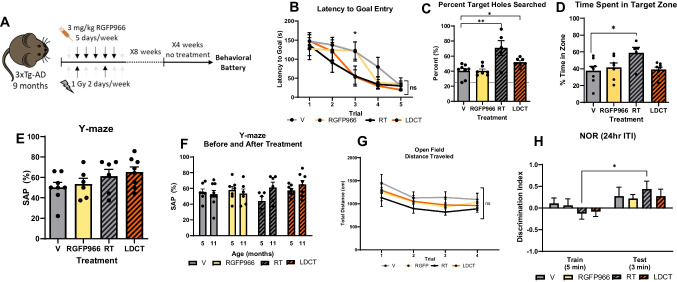
LDCT affects spatial memory of the 3xTg-AD mouse in the Barnes maze task of spatial learning. **A** Treatment schematic of aged 3xTg-AD mice. **B** Latency to first identification of target hole in Barnes maze. RT- and LDCT-treated mice learned to find hole faster than control-treated mice. All mice learned after 5 trials. **C** Proportion of target holes searched out of all holes searched during the probe trial 24 h after last training session. Only RT- and LDCT-treated mice performed significantly better than the control. **D** RT-treated mice spent larger proportion of total time spent in target zone during the probe trial 24 h after last training session. **E** Spontaneous alternation percent (SAP) in Y-maze of animals treated for 8 weeks. No significant differences were observed between treatment groups although RT and LDCT mice performed higher than V. **F.** Comparison of Y-maze SAP before and after treatment. No significance differences were observed although RT and LDCT animals performed better on average than they did before treatment. **G** No significant differences in distance traveled during open field habituation. **H** Discrimination index (DI) in NOR of treated animals during the training and testing phases (24 ITI). On average, all mice recognized the novel object in the testing phases; however, only RT-treated mice had a significantly improved DI compared to the training phase. Data are represented as mean ± SEM (*n* = 6–9). One-way ANOVA or two-way ANOVA followed by Dunnett’s or Holm-Sidak multiple comparisons test *p<0.05, **p<0.01

Based on our *in vitro* data suggesting low toxicity and complementary effects of LDCT with respect to anti-inflammatory response, amyloid processing, and the neurotrophic response, we progressed to *in vivo* studies in the triple transgenic (3xTg-AD) mouse model [44]. Aged, 9-month-old 3xTg-AD mice were treated for 8 weeks (*n* = 10/group) followed by a 4-week break in their home cages before behavioral assessment (Fig. 3A). We chose aged mice to mirror the late time point of therapeutic intervention at which clinical AD trials are often conducted where Aβ plaque formation and neurodegeneration have begun to occur [45]. Mice were treated daily with 3 mg/kg of RGFP966 (RGFP cohort) or 2X/week with 1 Gy cranial x-irradiation (RT cohort) with at least 48 h between RT doses. The LDCT cohort received both treatments with RGFP966 administration occurring immediately prior to RT, so the maximum brain penetrance of the drug occurred close to the time of RT. Vehicle-treated animals (V cohort) received vehicle and sham irradiation under isoflurane. Attrition occurred during treatment according with the expected lifespan of the 3xTg-AD model with no significant treatment-related deaths observed (Fig. S2A) [46, 47]. We waited 4 weeks after the completion of treatment before assessing memory to determine the durability of the effects on memory and behavior. In the Barnes maze (BM) test for spatial learning and memory, RT and LDCT mice demonstrated improved spatial learning in latency to goal after only three trials, whereas the RGFP and vehicle mice (V) required 5 total training trials to achieve a similar latency to goal time (Fig. 3B). In the BM test trial for spatial memory, the RT and LDCT cohorts successfully searched holes in the target quadrant 70.8% (*P*<0.01) and 51.8% (*P*<0.05) of the time, respectively, whereas V and RGFP cohorts had target rates of 40.3% and 40.5%, respectively (Fig. 3C). Alternatively, RT mice spent ~54% more total time searching in the target quadrant compared to V (Fig. 3D, *P*<0.05). No difference between groups was observed in latency to escape hole (Fig. S2A). No significant differences between groups or within treatment groups pre- and post-treatment were observed in the Y-maze (Fig. 3E, F). In the long-term novel object recognition (NOR) task (24 h intertrial interval [ITI]), only mice of the RT cohort appeared to recognize the novel object compared to baseline; however, between treatment groups, there was no significant difference based on the discrimination index (Fig. 3G, H). As an additional control, using a separate cohort of aged (18-month-old) wild-type C57Bl/6J mice, we confirmed that our RT protocol was not detrimental to memory as shown by no change to subthreshold recognition or location memory (Fig. S2C-H).

### Hippocampal Gene Expression Profiling Reveals Anti-AD Profile in LDCT Mice

**Fig. 4:**
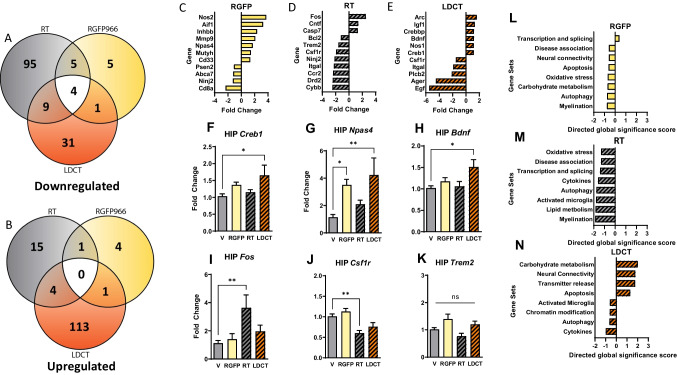
Neuropathology-focused transcriptomics reveal synergy of low-dose combination therapy in the 3xTg-AD hippocampus. **A**, **B** Venn diagrams of unique and shared differentially expressed genes (DEGs) in each treatment group show that LDCT elicits more uniquely upregulated gene changes and RT elicits more uniquely downregulated transcriptional changes. **C–E** Fold change of key significantly up and downregulated genes regarding neurotrophic signaling and neuroinflammatory pathways. **F–K** RT-qPCR validation of select NanoString DEGs. **L–N** Gene Set analysis reveals top enriched pathways within treatment groups. Data are represented as mean ± SEM (*n* = 6). One-way ANOVA using Dunnett’s multiple comparisons test and nCounter Advanced Analysis. *P<0.05, **P<0.01

NanoString gene expression analysis of 788 genes related to neuropathology using hippocampal RNA revealed the highest number of differentially expressed genes (DEGs) in the LDCT group followed by RT and then RGFP when normalized to vehicle-treated animals (Fig. 4A, B, Additional File 1). Notably, LDCT DEGs were mostly upregulated, and RT DEGs were mostly downregulated. Low-dose RGFP966 alone did not exhibit strong directionality or DEGs (Fig. S3A–C). Of the 788 genes analyzed, we highlighted the most significantly DEGs from each treatment group (Fig. 4C–E). DEGs of particular interest (*Creb1*, *Npas4*, *Bdnf*, *Fos*, *Csf1r*, and *Trem2*) were confirmed via RT-qPCR (Fig. 4F–K). NanoString Gene Set Analysis (related to Gene Ontology terms) of the LDCT cohort revealed a unique upregulation of genes involved in carbohydrate metabolism, neural connectivity, transmitter release, and apoptosis and downregulation of genes involved in activated microglia, chromatin modification, and cytokines, among others (Fig. 4N). This pattern of strongly up and downregulated Gene Sets is unique to the LDCT cohort, while RGFP and RT individually exhibited the strongest effects via downregulation of Gene Sets associated with oxidative stress, disease association, and myelination. (Fig. 4L, M and Fig. S4A). Interestingly, while genes associated with carbohydrate metabolism, neural connectivity, and apoptosis were downregulated by either individual treatment, we observed an upregulation when the treatments were combined in the LDCT cohort. Lastly, Cell Type Profiling which categorizes groups of genes based on cell-type enrichment revealed that RT exhibited its effects by downregulating microglia- and oligodendrocyte-enriched genes with negligible effects on astrocytes, neurons, and endothelial genes (Fig. S4B). In the LDCT cohort, only microglia-enriched genes were downregulated compared to the vehicle cohort, while RGFP966-treated animals did not exhibit any major cell type-specific alterations. Altogether, these data suggest that LDCT induces a unique, synergistic transcriptional environment that supports downregulation of innate immune response and upregulation of genes related to neural activity and memory.

### Radiotherapy and LDCT Reduce Brain Amyloid, P-Tau, and AD-Related Protein Pathology

**Fig. 5:**
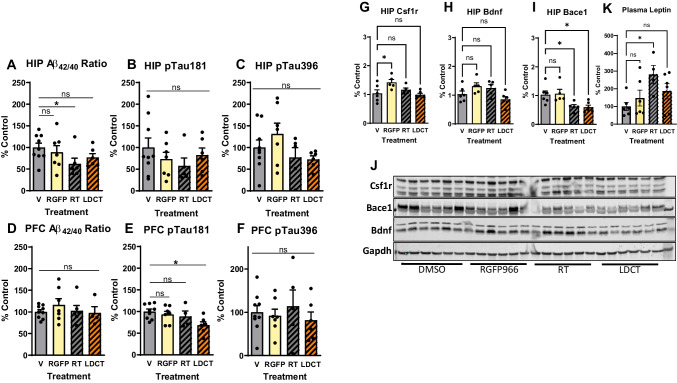
LDCT reduces Aβ_42/40_ ratio and pTau181 in hippocampus and prefrontal cortex, respectively. **A**, **D** ELISA reveals decrease in HIP Aβ_42/40_ ratio in RT-treated animals but not in PFC or other treatment groups. **B**, **E** ELISA reveals decreased pTau181 in the PFC of LDCT-treated animals but not in HIP or other treatment groups. **C**, **F** No change in pTau396 was observed via ELISA in either PFC or HIP. **G–J** Immunoblotting and quantification for Csf1r (**G**), Bdnf (**H**), and Bace1 (**I**) show a decrease in Bace1 protein expression in RT- and LDCT-treated groups. **K** Luminex assay in 3xTg-AD plasma reveals increase in leptin in RT-treated mice, potentially a biomarker for therapeutic RT dosing. Data are represented as mean ± SEM (*n* = 5–9). One-way ANOVA using Dunnett’s multiple comparisons test *p<0.05

To investigate the effects of LDCT on amyloid pathology, we measured soluble Aβ_40_ and Aβ_42_ in the hippocampus (HIP) and prefrontal cortex (PFC) using ELISA assays (Table 1). RT significantly reduced (38%, *P*<0.05) the Aβ_42/40_ ratio in the HIP but not in the PFC compared to the V cohort (Fig. 5A, D). ELISA assays were also performed to assess soluble p-tau at threonine 181 (p-tau181) and serine 396 (p-tau396). P-tau181 is one of the most reliable AD biomarkers of disease status in plasma and correlates with the presence of hyperphosphorylated neurofibrillary tau tangles in the brain [48–50]. LDCT resulted in a 25% reduction in p-tau181 (*P*<0.05) in the PFC compared to V mice, while no change in HIP nor PFC p-tau396 was observed (Fig. 5B–F). We then performed western blotting in hippocampal lysates to assess continuity of gene and protein expression data and observed no change in Csf1r or Bdnf expression (Fig. 5G, H). However, Bace1 was downregulated by RT and LDCT (1.64-fold, *P*<0.05 and 1.69-fold, *P*<0.05, respectively) (Fig. 5I). Lastly, we used multiplex Luminex technology (see Methods and Materials) to profile the peripheral immune response in treated 3xTg-AD plasma. A total of 48 cytokines, chemokines, and growth factors were assessed. In our cohort, only the hormone leptin was significantly upregulated (2.8-fold, *P*<0.05) in RT plasma (Fig. 5K) compared to the V cohort; however, mRNA expression of leptin in brain or liver tissue could not be detected (data not shown).

**Table 1:** Dilution factors used for soluble protein in ELISAs

Dilutions	Aβ_42_	Ultrasensitive Aβ_42_	Aβ_40_	p-tau181	p-tau396	Total tau
PFC	n/a	8.75X	10X	20X	15X	5000X
HIP	10X	n/a	10X	400X	30X	50000X
Media	10X	n/a	100X	n/a	n/a	n/a

## Discussion

We hypothesized that HDAC3 inhibition combined with low-dose cranial radiotherapy has the potential to synergistically reduce microglial activation, induce neurotrophic gene expression, preserve memory, and ameliorate common AD protein pathologies. Our *in vitro* results revealed that RGFP966 and RT act complementarily to create a tolerant immune phenotype in murine microglia and shift APP processing to the non-amyloidogenic pathway. Most notably, LDCT in 3xTg-AD mice synergistically created a unique, anti-AD gene expression profile including downregulation of genes related to activated microglia and upregulation of those related to neural connectivity. These in vivo gene expression findings were also accompanied by improvements in spatial memory and reduction of Aβ_42/40_ ratio and pTau181 in the brains of cohorts receiving RT or LDCT, respectively. While our results do not unanimously demonstrate synergy across all assays, the data presented for individual treatments and LDCT provide novel, mechanistic insight into multitarget approaches to ameliorate AD pathology.

Microglia are the dominant immune cells of the brain with the potential to exacerbate or alleviate AD pathology, depending on disease stage and other external stressors [51–53]. Individually, both RT and HDAC3 inhibition at higher doses have been shown to exhibit anti-inflammatory properties in innate immune cell populations, namely in microglia [54–57]. Microglia-enriched genes including *Spi1*, *Trem2*, *Ager*, and *Csf1r*—all investigated in the present studies—are critical regulators of microglial activation and viability [58–61]. Furthermore, it is well established that depletion of microglia in the brain of AD mouse models via Csf1r inhibition ameliorates AD pathology, improves memory, and is now being investigated in the clinic (NCT04121208) [62–64]. In support of this, our *in vivo* investigation of LDCT revealed downregulation of *Csf1r* and *Ager* in the HIP, but not *Trem2*, although *Trem2* was found consistently downregulated by RT in both our in vitro and in vivo data*.* TREM2 is considered a microglial pivot point with the ability to govern microglial polarization states via lipid metabolism, phagocytic capacity, cytokine production, and many other phenotypes [65]. While immunotherapies and oligonucleotides targeting TREM2 activation and expression are major neuroinflammatory drug candidates in the next wave of potential AD therapies, it has become evident that TREM2 activity is context-dependent (i.e., disease state, tissue, and cell type). In fact, there is strong evidence supporting both the upregulation and downregulation of Trem2 in preclinical AD models [66, 67], revealing that the role of microglial Trem2 is not as straightforward a target for AD as previously thought [68–70]. Our data suggest that LDCT of RGFP966 and cranial RT achieves overall downregulation of microglial activation without significantly affecting the homeostatic role of *Trem2*, potentially achieving a higher benefit/risk ratio than either treatment alone in the context of neuroinflammation.

Preclinical efforts to upregulate neurotrophic gene expression programs related to synaptic plasticity have been encouraging, and synapse functionality correlates tightly with cognitive function in AD [71, 72]. While prior research and our behavior data may suggest that either RT or RGFP966 at higher doses holds potential on their own in this respect, our gene expression data herein supports the existence of a molecular synergy between the two low-dose therapies that includes simultaneous upregulation of neurotrophic genes and downregulation in neuroimmune genes. Notably, Gene Set analysis (NanoString) of our data revealed that the neural connectivity pathway was significantly upregulated only in the LDCT group, including *Bdnf* and the neurotrophic immediate early gene (IEG) *Arc*. Our in vitro and in vivo results assessing gene expression of neurotrophic genes *c-Fos*, *Npas4*, and *Bdnf* also support this synergy hypothesis. HDAC3 is a negative regulator of Npas4-mediated *Bdnf* gene expression [73]. Furthermore, Titus et al. showed that HDAC3 does not regulate expression of the IEG *c-Fos* at its promoter region, a transcription factor that also regulates *Bdnf* expression [74]. Accordingly, we observed *Npas4* upregulation in the RGFP966-treated group and upregulated *c-Fos* expression only in the RT-treated group (Fig. 4G, I), with *Npas4* and *Bdnf* either more significantly or uniquely upregulated in the LDCT group, respectively. Combined with RT, RGFP966 induced larger and/or more significant increases in neurotrophic genes (Fig. 2B and Fig. 4E). Our data show that modulating relevant pathways rather than individual AD-associated targets with low-dose, minimally invasive treatments can ameliorate AD pathology and improve memory.

In many promising clinical AD studies including those for acetylcholinesterase inhibitors and non-pharmacological modulators of neural activity, cognitive improvements disappear shortly after the treatment course is completed [75, 76]. While any clinically meaningful improvement in cognition is valuable, the ideal AD therapeutic should be aimed at potentially curative, long-lasting effects and not simply disease maintenance. Accordingly, our delayed memory assessment in 12-month-old 3xTg-AD mice 1 month after the termination of 2 months of combination therapy revealed sustained improvements in spatial memory in this mouse model for RT and to a lesser extent, LDCT-treated animals. This was encouraging given the small cohort size and memory testing in mice of such an advanced stage. This study is one of the few to assess the sustained effects of a preclinical intervention after treatment completion, especially cognitive performance. However, it is important to note that our study only analyzed *in vivo* data at a single timepoint post-treatment. It would be valuable for follow-up studies to assess memory and molecular profiles at multiple timepoints after treatment completion in order to establish the onset and longevity of perceived benefits.

The two previous studies examining the delayed effects of RT on cognition by Marples et al*.* (2016) and Kim et al. (2020) also observed improved spatial memory in the Morris water maze task 8 weeks after treatment completion [22, 23]. A third study by Wilson et al. (2020) did not assess memory but performed 8-week delayed immunohistochemistry detection of hippocampal amyloid plaque load and tau hyperphosphorylation, observing RT-induced reduction in both [77]. All three of these studies used a treatment regimen of 5 consecutive daily fractions of 2 Gy, although the study by Marples et al. investigated an additional range of dosage regimens for plaque reduction. Similar to the present study, Kim et al. examined the effects of low-dose x-irradiation (LDIR) in cultured microglia and observed similar findings as reported here in regard to the ability of LDIR to reduce the expression of IL1β and IL6 in response to LPS, an inflammatory stimulus. Our work aligns well with this study and another molecular study by Kim et al. showing the ability of RT to dramatically affect the microglial phenotype in the short- and long-term [21]. However, we are the first group to suggest that RT can be used to synergize with other anti-AD or cognitive-enhancing drugs at subthreshold doses to achieve sustained reduction in neuroinflammation and improved cognition and neurotrophic gene expression.

The radiation dose response curve is biphasic, consistent with the principal of hormesis where low concentrations of a potential toxin stimulate rather than overburden cellular repair mechanisms [78]. Defining the low- versus high-dose threshold for anti-inflammatory activity, anti-amyloidogenic APP processing, and neurotrophic stimulatory potential of RT remains a high priority in preclinical and clinical studies. From the Cell Type Profiling data obtained from our 3xTg-AD animals treated with 1 Gy X 16 fractions over 2 months, we observe that RT exhibits the strongest effects on microglia which have well-established roles in modulating amyloid deposition and clearance [70, 79, 80]. Data from Marples et al. using hemibrain irradiation (2 Gy X 10 daily fractions) also demonstrates downregulation of immune-related microglia targets *Iba1*, *Mip-2*, and *Inf-γ* [22]. This suggests that hemibrain RT can be effective through localized downregulation of the microglial immune response. Such an anti-inflammatory effect from localized brain RT holds clinical promise, especially considering the potential of low-dose synergy when combined with a cogntive enhancing or disease modifying small molecule.

Notably, we did not notice behavioral side effects due to any of the low-dose treatments when assessing locomotion and survival. RGFP966 was non-toxic to murine microglial cells up to 10 μM compared to M344 and quisinostat which are both pan-HDACi that have been investigated for their potential to improve cognition or cross the BBB, respectively (Fig. S1H). This could be due to the selectivity for HDAC3 over other class I and IIb HDACs or possibly the class of the HDACi tested (e.g., benzamide vs hydroxamic acid-derived) [81, 82]. Pan-HDAC inhibition is generally toxic, inducing various cytopenias, and is the cause for continued isoform-specific investigation of HDACs in neurodegenerative diseases [83]. Additionally, RGFP966 has a relatively short half-life in the brain with penetrance peaking 15–30 min after administration and declining quickly over the next 2 h [84]. Similar to our previous work with M344 in 3xTg-AD mice, we and others propose that this short “pulse” effect in the brain may be enough to obtain therapeutic benefits while possibly avoiding side effects [8, 85]. The work presented here supports combining distinct but complementary anti-AD approaches to maximize therapeutic benefit while minimizing side effects that often accompany small molecule and radiation therapies, a cognitive and molecular “goldilocks effect.” Indeed, combination therapies have successfully become standard of care in many other disease contexts, especially chronic diseases of aging with multiple pathologies and polygenic risk contributors [86–88].

## Conclusions

Taken together, the data presented here support 3 main findings: (1) low-dose HDAC3i and radiotherapy have both unique and synergistic anti-inflammatory effects on microglia in vitro, (2) LDCT induces a unique anti-AD gene expression profile in vivo that includes downregulation of microglial genes and upregulation of neurotrophic genes, and (3) RT and LDCT improve memory in aged 3xTg-AD mice. Physical and pharmacological combination therapies are underexplored in AD, yet it is evident that patients diagnosed with AD and other multifactorial chronic diseases of aging may benefit from a multipronged approach.

## Supplementary Information

ESM 1 **Supplementary Figure 1. A-G.** Time course RT-qPCR of microglial enriched genes in LDCT treated SIM-A9 cells. 48hr data are presented in main Figure 1. **H.** toxicity profile of RGFP966 against 2 other pan-HDACi in SIM-A9 cells using Cell Titer-Glo. **I.** ELISA of conditioned media used in experiments presented in Fig. 1C-F collected from APP_Swe/Ind_ transiently transfected murine N2A cells. Data are represented as mean ± SEM (RT-qPCR: n=6 biological replicates, Cell TiterGlo n=3). A-G: two-way ANOVA with Dunnett’s multiple comparison test *P<0.05, **P<0.01, ***P<0.001, ****P<0.0001 compared to Vehicle. # denotes significance in comparison to RT. % denotes significance in comparison to RGFP966. (PDF 195 kb)

ESM 2 **Supplementary Figure 2. A.** Kaplan-Meier curve showing no significant difference in treatment effects on survivorship. **B.** Additional Barnes maze data showing so significant difference in latency to first identification of goal between treatment groups after 5 training trials (n=6-9). **C-H.** 18-month-old C57Bl/6J mice received 8 weeks of RT therapy identical to 3xTg-AD mice (2 Gy/week in 1 Gy doses). Subthreshold recognition and location memory were tested along with working memory in Y-maze. No significant differences were observed between sham and irradiated animals. **I.** Heat map of 3xTg-AD mice during final probe trial in Barnes maze demonstrating that RT mice on average spent more time in target quadrant (bottom left) than elsewhere in the maze. Data are represented as mean ± SEM (n=5-9). Student’s t-test or one-way ANOVA using Dunnett’s multiple comparisons test. (PDF 257 kb)

ESM 3 **Supplementary Figure 3: A-C.** Volcano plots of HIP DEGs in each treatment group normalized to vehicle-treated cohort. (PDF 119 kb)

ESM 4 **Supplementary Figure 4: A.** NanoString Genes Set analysis of DEGs in each treatment group. Note that LDCT and RT cohorts have an opposing expression signature while RGFP alone does not elicit as strong of a transcriptional response. This unique LDCT signature suggests that the treatments favorably interact *in vivo* to promote neurotrophic signaling and dampen microglial activation. **B.** NanoString cell type analysis performed on bulk HIP RNA shows that RT alone strongly down regulates genes enriched in microglia, neurons, astrocytes, and oligodendrocytes compared to control treated animals. This strong downregulation is not observed in RGFP treated animals and only microglia-enriched genes are modestly downregulated while neuron-enriched genes are modestly upregulated in the LDCT HIP. (PDF 129 kb)

ESM 5 Additional File 1.xls: Normalized nanostring data from 2 month-treated 3xTg-AD hippocampus (XLSX 30 kb)

## Data Availability

All data generated or analyzed during this study are included in this published article and its supplementary information files or available through NCBI’s Gene Expression Omnibus.

## Abbreviations

3xTg-AD: triple transgenic AD mouse
AD: Alzheimer’s disease
ADAM10: ADAM Metallopeptidase Domain 10
Ager: Advanced Glycosylation End-Product Specific Receptor
ANXV: Annexin V
Apoe: Apolipoprotein E
APP: amyloid precursor protein
Aβ: amyloid-beta
BACE1: beta-site amyloid precursor protein (APP) cleaving enzyme-1
BDNF: brain-derived neurotrophic factor
CD68: Cluster of Differentiation 68
Creb1: CAMP Responsive Element Binding Protein 1
Csf1r: receptor of the colony-stimulating factor-1
Fos: Fos Proto-Oncogene, AP-1 Transcription Factor Subunit
GFAP: Glial fibrillary acidic protein
HDAC: histone deacetylase
HDAC3: histone deacetylase 3
HDACi: HDAC inhibitor
HIP: hippocampus
Iba1: Ionized calcium binding adaptor molecule 1
Il10: interleukin-10
Il1b: interleukin-1beta
Il6: Interleukin-6
LDCT: low-dose combination therapy
NPAS4: Neuronal PAS Domain Protein 4
PFC: prefrotal cortex
PSEN1/2: Presenilin 1/2
p-tau: phosphorylated tau
RT: radiotherapy
sAPPα: soluble APP alpha cleavage fragment
Spi1: spleen focus forming virus (SFFV) proviral integration oncogene
Tnfα: tumor necrosis factor alpha
Trem2: Triggering Receptor Expressed On Myeloid Cells 2
V: vehicle
