# Supplementary figures and images for: Investigating the Synergistic Potential of Low-Dose HDAC3 Inhibition and Radiotherapy in Alzheimer’s Disease Models
Source: Mol Neurobiol. 2023 May 12;60(8):4811–27. doi: 10.1007/s12035-023-03373-0 (PMC10293392; doi:10.1007/s12035-023-03373-0)

Figure S1

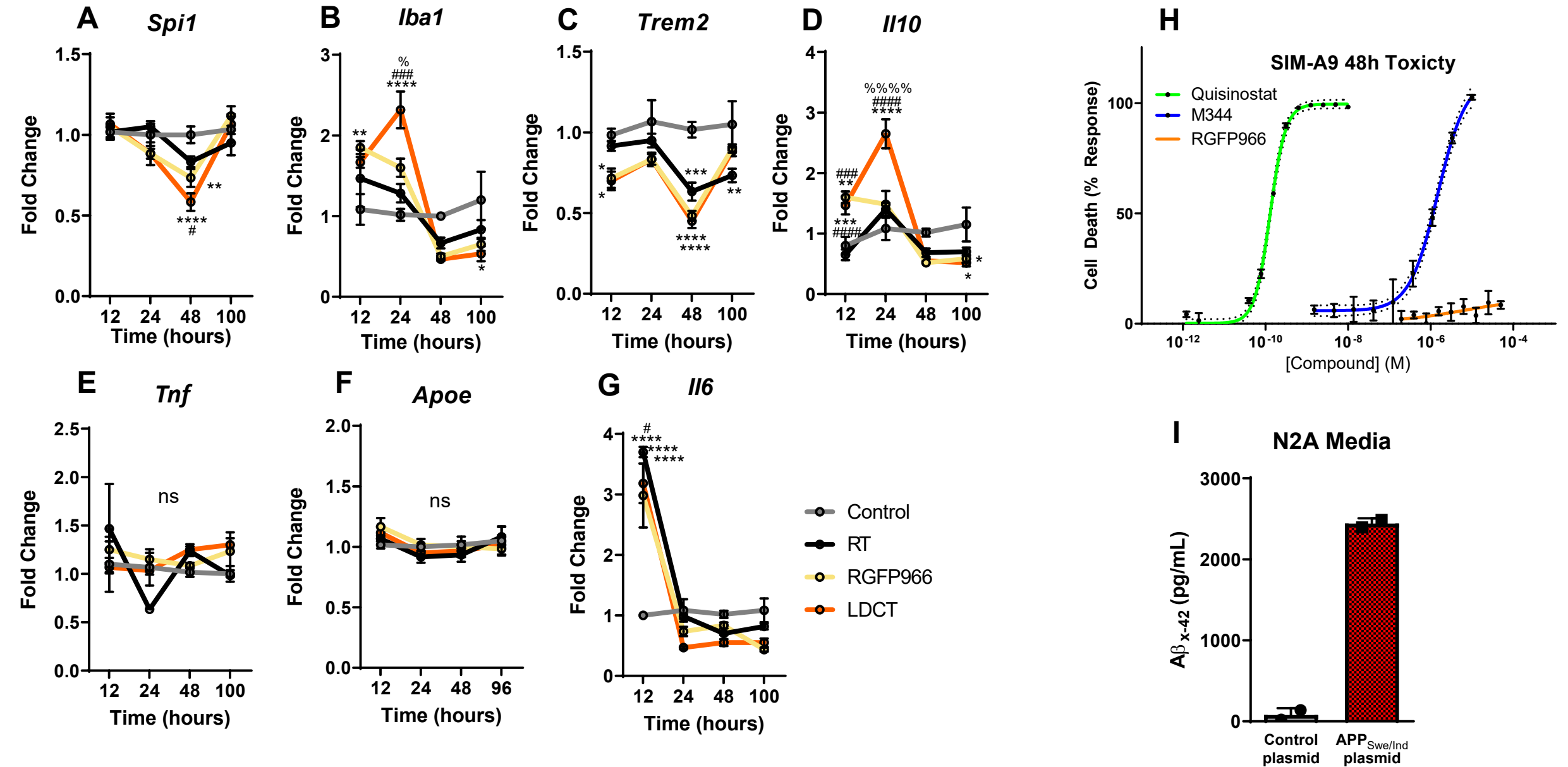

Supplement: Supplementary file 1 — Supplementary Figure 1. A-G. Time course RT-qPCR of microglial enriched genes in LDCT treated SIM-A9 cells. 48hr data are presented in main Figure 1. H. toxicity profile of RGFP966 against 2 other pan-HDACi in SIM-A9 cells using Cell Titer-Glo. I. ELISA of conditioned media used in experiments presented in Fig. 1C-F collected from APPSwe/Ind transiently transfected murine N2A cells. Data are represented as mean ± SEM (RT-qPCR: n=6 biological replicates, Cell TiterGlo n=3). A-G: two-way ANOVA with Dunnett’s multiple comparison test *P<0.05, **P<0.01, ***P<0.001, ****P<0.0001 compared to Vehicle. # denotes significance in comparison to RT. % denotes significance in comparison to RGFP966. (PDF 195 kb) [file 12035_2023_3373_MOESM1_ESM.pdf]

Figure S2:

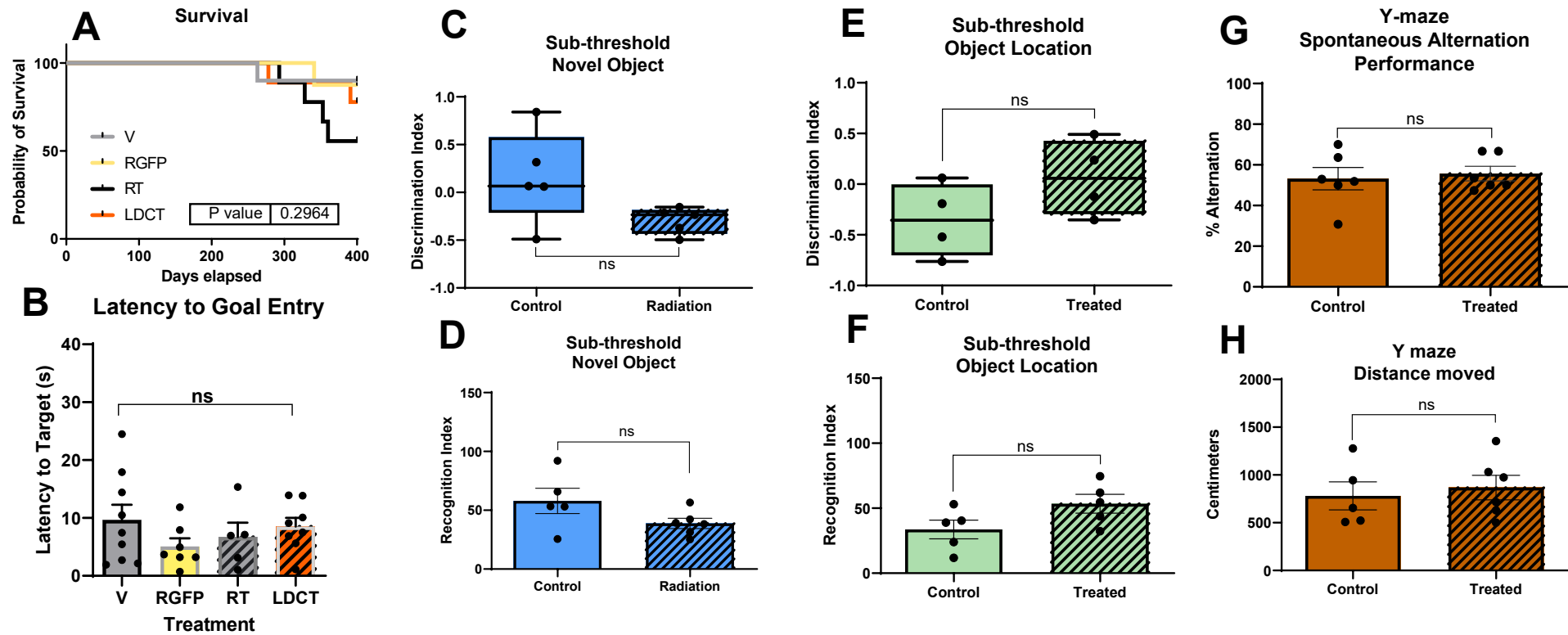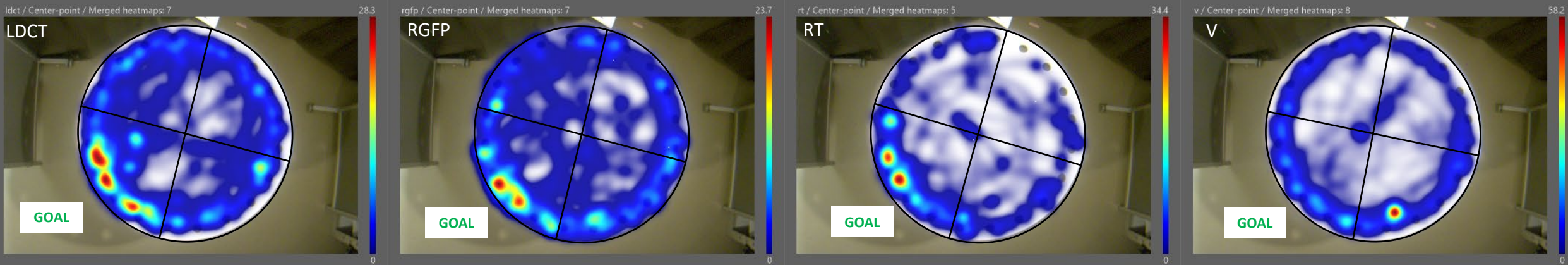

Supplement: Supplementary file 2 — Supplementary Figure 2. A. Kaplan-Meier curve showing no significant difference in treatment effects on survivorship. B. Additional Barnes maze data showing so significant difference in latency to first identification of goal between treatment groups after 5 training trials (n=6-9). C-H. 18-month-old C57Bl/6J mice received 8 weeks of RT therapy identical to 3xTg-AD mice (2 Gy/week in 1 Gy doses). Subthreshold recognition and location memory were tested along with working memory in Y-maze. No significant differences were observed between sham and irradiated animals. I. Heat map of 3xTg-AD mice during final probe trial in Barnes maze demonstrating that RT mice on average spent more time in target quadrant (bottom left) than elsewhere in the maze. Data are represented as mean ± SEM (n=5-9). Student’s t-test or one-way ANOVA using Dunnett’s multiple comparisons test. (PDF 257 kb) [file 12035_2023_3373_MOESM2_ESM.pdf]

Figure S3

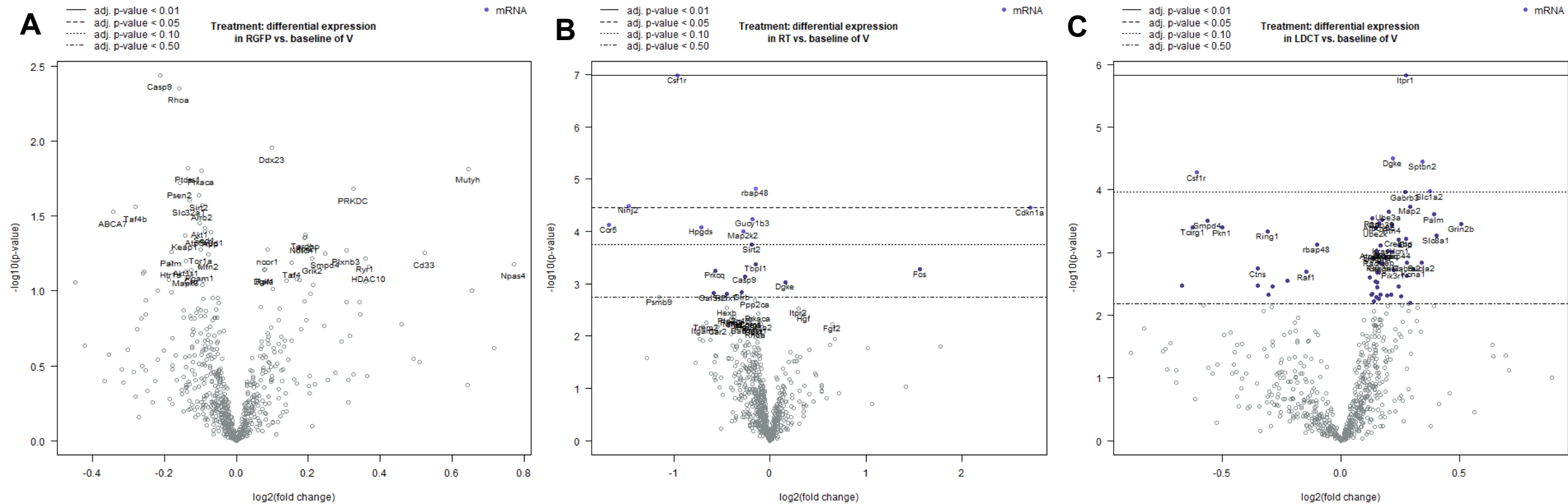

Supplement: Supplementary file 3 — Supplementary Figure 3: A-C. Volcano plots of HIP DEGs in each treatment group normalized to vehicle-treated cohort. (PDF 119 kb) [file 12035_2023_3373_MOESM3_ESM.pdf]

Figure S4

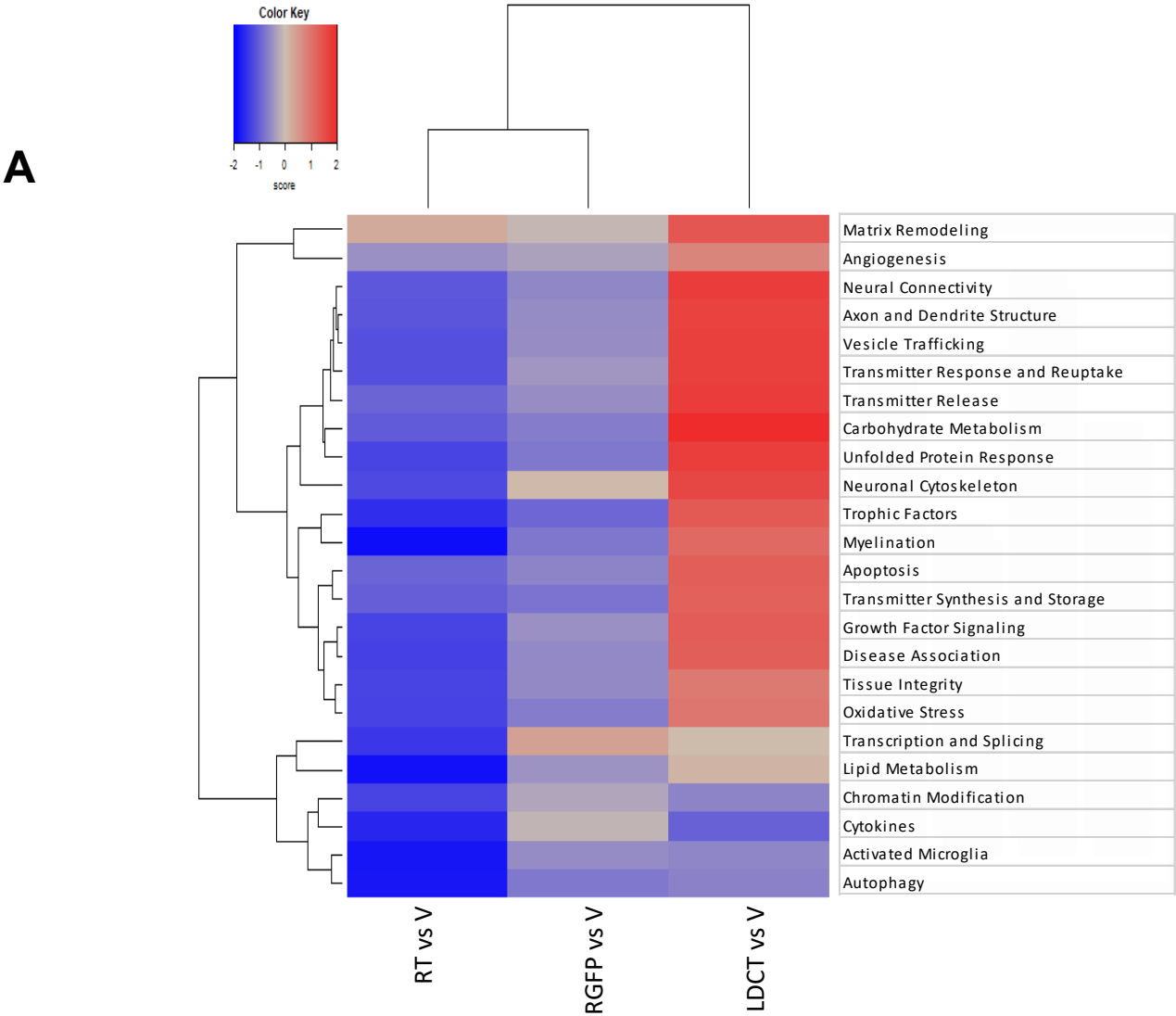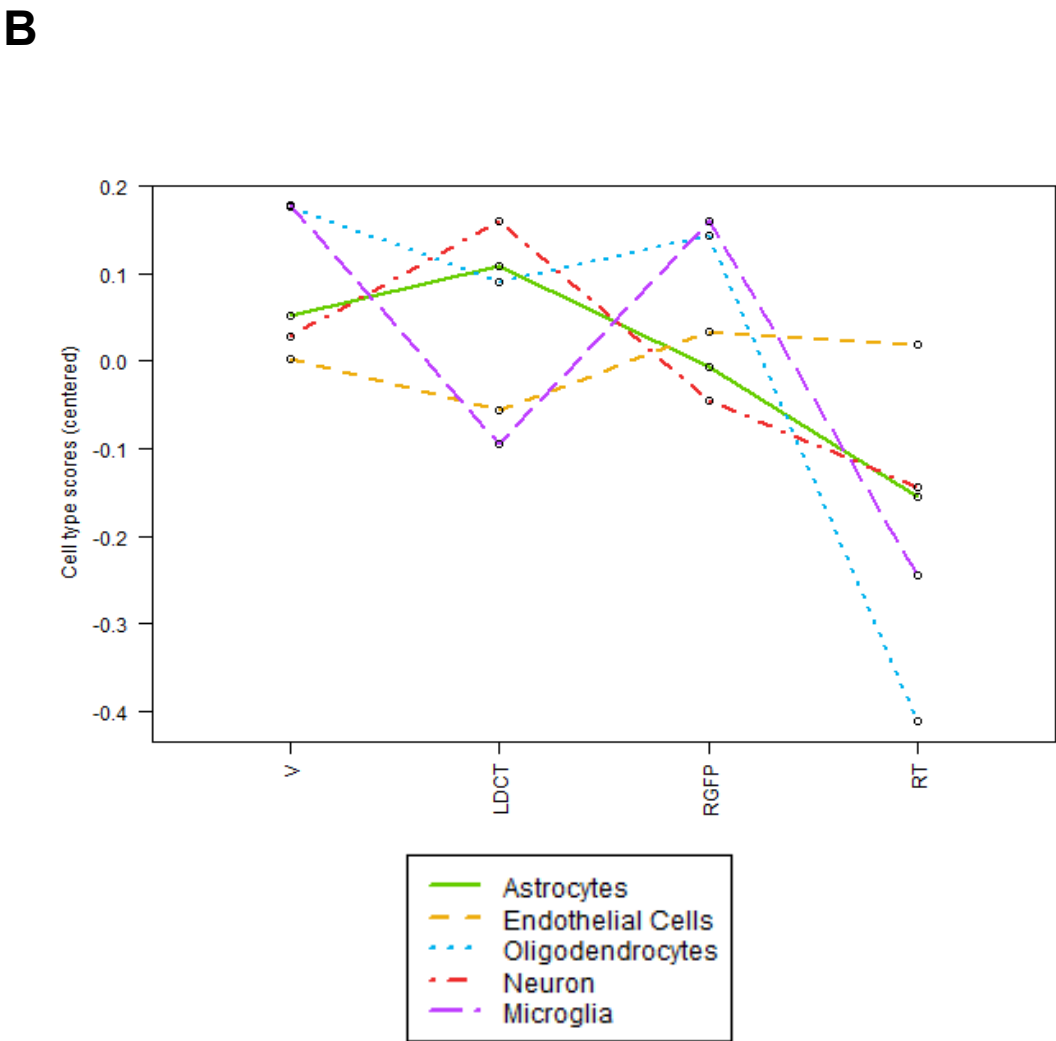

Supplement: Supplementary file 4 — Supplementary Figure 4: A. NanoString Genes Set analysis of DEGs in each treatment group. Note that LDCT and RT cohorts have an opposing expression signature while RGFP alone does not elicit as strong of a transcriptional response. This unique LDCT signature suggests that the treatments favorably interact in vivo to promote neurotrophic signaling and dampen microglial activation. B. NanoString cell type analysis performed on bulk HIP RNA shows that RT alone strongly down regulates genes enriched in microglia, neurons, astrocytes, and oligodendrocytes compared to control treated animals. This strong downregulation is not observed in RGFP treated animals and only microglia-enriched genes are modestly downregulated while neuron-enriched genes are modestly upregulated in the LDCT HIP. (PDF 129 kb) [file 12035_2023_3373_MOESM4_ESM.pdf]
